# Safety of Simparica Trio^®^ (sarolaner, pyrantel, moxidectin) in heartworm-infected dogs

**DOI:** 10.1186/s13071-023-05700-9

**Published:** 2023-03-31

**Authors:** Sheerin Mathur, Phyllis B. Malpas, Sean Mahabir, Joseph Boucher, Aleah Pullins, Genevieve Gagnon, Tom L. McTier, Steven Maeder

**Affiliations:** Zoetis, Veterinary Medicine Research and Development, Michigan, 49007 USA

**Keywords:** Simparica Trio, Moxidectin, Safety, Heartworm disease, *Dirofilaria immitis*, Dogs, Microfilaremia, Pharmacokinetics, PK

## Abstract

**Background:**

Assessment of the safety of heartworm preventatives in dogs with pre-existing patent heartworm (*Dirofilaria immitis*) infections is necessary because rapid adult worm and microfilarial death can lead to severe clinical complications, including thromboembolism and anaphylactic shock in dogs. The aim of this study was to determine the clinical safety of Simparica Trio^®^ (sarolaner, pyrantel, moxidectin) in heartworm-infected dogs and the degree of microfilaricidal and adulticidal activity of three consecutive monthly treatments of Simparica Trio.

**Methods:**

Twenty-four laboratory Beagle dogs were implanted with 10 male and 10 female *D. immitis* (ZoeKY isolate), and once infection was patent, they were randomized equally among three groups to receive no treatment, 1× or 3× the maximum recommended label dose of Simparica Trio. Dogs in the treated groups received Simparica Trio on days 0, 28 and 56. In-life assessments included body weight, physical examinations, clinical observations, daily general health observations, a quantitative estimate of food consumption and blood collections for pharmacokinetic (PK) analysis, microfilariae (MF) counts and *D. immitis* antigen testing. At the end of the study the heart, lungs and pleural and peritoneal cavities were examined for adult *D. immitis* worms.

**Results:**

Simparica Trio was generally well tolerated. Emesis occurred at low frequency in all groups including control. Abnormal stool occurred occasionally in the 1× and 3× groups throughout the 3-month study. Fever (> 104 °F/40 °C) was recorded in one 1× and one 3× dog 1 day after the first dose and resolved by the following day. No severe hypersensitivity reactions occurred. The mean number of circulating microfilariae (MF) counts in the control group increased from 12,000/ml at study start (Day 0) to > 20,000/ml at Day 28 and remained > 20,000/ml for the duration of the study. The least squares means of circulating MF were reduced by 69.8% on Day 1 and 97.4% on Day 7 for the 1× group and remained at > 99% lower than the control group for the remainder of the study. Similarly, least squares means of circulating MF were reduced by 85.3% on Day 1 and 93.9% on Day 7 for the 3× group and remained > 98% lower than the control group for the remainder of the study. At the end of the study, the mean number of implanted adult worms recovered was < 10 per sex in all groups with 90%, 85% and 75% of live adult heartworms recovered in control, 1× and 3× treatment groups, respectively. Low numbers of dead adult worms were recovered in 1× and 3×, with none in control. Following each dose, the moxidectin and sarolaner AUC and C_max_ had close to dose proportional increases.

**Conclusions:**

This study demonstrated that Simparica Trio (sarolaner, pyrantel, moxidectin) was well tolerated when administered to heartworm-positive dogs at 1× and 3× the maximum recommended dose at 28-day intervals for 3 consecutive months. Simparica Trio significantly reduced microfilaria counts in both treatment groups, without significant clinical consequences. At the doses administered, Simparica Trio had minor adulticidal activity but resulted in no clinical sequelae.

**Graphical Abstract:**

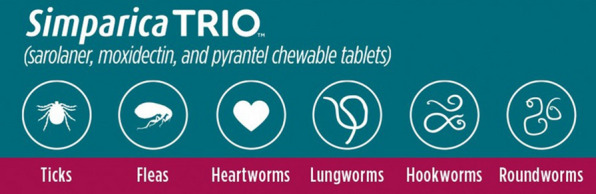

## Background

Simparica Trio (Zoetis) is the first combination endectocide containing an isoxazoline (sarolaner), moxidectin and pyrantel licensed in the USA that prevents heartworm disease (*Dirofilaria immitis*) and treats and controls fleas, ticks and intestinal nematodes. Simparica Trio is a chewable flavored tablet with a minimum label dosage of 1.2 mg/kg sarolaner, 5 mg/kg pyrantel (as pamoate salt) and 24 µg/kg moxidectin. Other licensed combination products contain the macrocylcic lactone (ML) ivermectin, moxidectin or milbemycin oxime for canine heartworm disease prevention. Among MLs, moxidectin has some unique attributes such as high potency, long half-life, wide therapeutic index and versatility in formulation that could help in overcoming heartworm drug resistance [1]. The dose of moxidectin included in Simparica Trio was optimized to increase the effectiveness against known drug-resistant heartworm isolates [2].

Macrocyclic lactones (ivermectin, selamectin, milbemycin oxime, moxidectin) target the L3 (infective) and L4 stages of *D. immitis* in the subcutaneous tissues. While this class of drug is most potent against the L3 and L4 stages, MLs at the doses used for HW prevention can also affect both microfilariae (MF) and adult worms [3]. These effects vary with the specific ML, dose of the ML and number of consecutive doses administered for monthly products or the length of time after treatment for the extended-release injectable products. MLs in combination with doxycycline can have additional adulticidal effects over time [4, 5]. Furthermore, MLs are considered generally safe for use in dogs harboring existing adult HW infections with circulating MF unlike the previously licensed daily oral heartworm preventives containing diethylcarbamazine [6]. This attribute of MLs makes preventing new heartworm infections during melarsomine (Immiticide^®^/Diroban^®^) adulticidal heartworm treatment possible. The American Heartworm Society (AHS) recommends that “A macrocyclic lactone preventive should be administered for 2 months prior to administering melarsomine to reduce new infections and eliminate existing susceptible larvae” [7]. If dogs are still positive for microfilariae by Day 120 of treatment, the AHS recommends administering a microfilaricide. Currently, only one moxidectin-containing product is approved as a heartworm microfilaricide (Advantage Multi® Bayer); however, all MLs using label and extra-label doses at varying frequencies of dosages have demonstrated microfilaricidal effects [3].

It is necessary to evaluate any new heartworm disease preventive products for safety in HW-positive dogs before product approval to ensure their safe use in heartworm-positive dogs in the general population. The purpose of the current investigation was to evaluate the safety of the maximum therapeutic dose on the product label of Simparica Trio [2.4 mg/kg of sarolaner + 10 mg/kg pyrantel + 48 μg/kg (0.048 mg/kg) moxidectin] in dogs with laboratory-induced adult *D. immitis* infections and with circulating MF.

## Methods

### Animals

A total of 24 purpose-bred laboratory Beagle dogs, including 12 males and 12 females approximately 10 months of age, were enrolled in the study. Dogs were housed individually in environmentally controlled indoor runs with ad libitum access to food and water.

Approximately 4 months prior to study initiation, dogs were surgically transplanted with 10 male and 10 female *D. immitis* worms (ZoeKY isolate), a known ML-susceptible isolate [8]. Prior to the study initiation, infection status was verified by a *D. immitis* antigen test (DiroCHEK^®^ Heartworm Antigen Test Kit, Zoetis, Parsippany,NJ), and blood samples were verified to contain > 500 circulating microfilariae (MF) per ml by modified Knott’s method. No animal had previous exposure to a macrocyclic lactone.

### Study design

This study was conducted in compliance with requirements of US FDA GLP regulations [9]. The study aligned with recommendations of the “VICH GL 43 (Target Animal Safety)-Pharmaceuticals [10].” To control bias, treatment allocation was not disclosed to any person responsible for conducting subjective observations. This study was also conducted in compliance with the Animal Welfare Act of 1966 (and subsequent amendments including the Animal Welfare Acts of 1970, 1976, and 1985) for the care of animals. The study procedures and care and use of animals were reviewed and approved in advance of the study by test facility Institutional Animal Care and Use Committee.

The 24 dogs were randomized to treatments (8 per group) according to split-plot design within room, with sex as the whole plot factor and treatment as the split-plot factor. Animals were blocked by sex and microfilariae (MF) count and randomized within blocks of size 3 and assigned to treatment groups: negative control (empty capsule), 1× and 3× of the maximum recommended label dose of Simparica Trio. Dogs in the treated groups received Simparica Trio on days 0, 28 and 56 administered as whole tablets (Table 1). The recommended minimum dosage for the product is 1.2 mg/kg sarolaner + 5 mg/kg pyrantel + 24 μg/kg (0.024 mg/kg) moxidectin. However, per VICH GL 43, the margin of safety is assessed based on multiples of the maximum recommended therapeutic dose (MRTD), which is the dose intended for administration to the lightest-weight dog in the widest dose band. For Simparica Trio, the MRTD is 2.4 mg/kg of sarolaner + 10 mg/kg pyrantel + 48 μg/kg (0.048 mg/kg) moxidectin. The doses were based on the most recent individual body weight. Because point dosing of the calculated MRTD is not possible with whole tablets, doses were rounded up using the smallest tablet size.

**Table 1:** Margin of safety study design of Simparica Trio in heartworm-infected (*Dirofilaria immitis*) Beagle dogs

Treatment group	Treatment	Dose level	# Dogs (M/F)	Dosing days	Necropsy day
Control^a^	Placebo	0	8 (4/4)	0, 28, and 56	84
1×^b^	Sarolaner	2.4 mg/kg	8 (4/4)		
	Pyrantel	10 mg/kg			
	Moxidectin	48 µg/kg			
3×^b^	Sarolaner	7.2 mg/kg	8 (4/4)		
	Pyrantel	30 mg/kg			
	Moxidectin	144 µg/kg			

^a^Empty HPMC capsules for oral administration

^b^Maximum dose from the dose banding

In-life assessments included weekly body weight measurements, veterinary physical examinations, veterinary clinical observations, technician-led daily general health observations and a daily quantitative estimate of food consumption. Blood collections for pharmacokinetic (PK) analysis were performed pre-dose and 2, 8, 24, 72, 168, 336, 504 and 672 h after each dose. Moxidectin, sarolaner and pyrantel plasma concentrations were measured using validated LC-MS/MS methods. Blood was also collected for heartworm testing throughout the study as follows: MF counts on Days -14, -8, 0 (pre-dose), 1, 3, 7, 14, 21, 28 (pre-dose), 35, 56 (pre-dose), 63 and 83 and *D. immitis* antigen testing Day -14 and pre-dose on days 28, 56 and 83.

Dogs were humanely killed after an intravenous injection of heparin to facilitate parasite recovery via an approved pentobarbital euthanasia solution on Day 84. After euthanasia, the pleural and peritoneal cavities were examined for adult *D. immitis* worms, and the posterior and anterior venae cavae were clamped before removal of the heart and lungs. The pre-cava, right atrium, right ventricle and pulmonary arteries (including those coursing through the lungs) were dissected and examined for worms. The worms from each dog were counted, identified as adult male or female and classified as either dead or alive based on motility and appearance [11]).

### Data analysis

Observational data (general health observations, clinical observations and physical examinations) were summarized with frequency distributions by treatment and time point. Body temperature, body weight, weekly average food consumption, adult heartworm counts and MF counts were summarized with descriptive statistics. Body weight, weekly average food consumption and MF counts were statistically analyzed using a general linear mixed model for repeated measures including fixed effects of treatment, time, sex and all interactions among those effects. The random effects included room, block within room and sex, the interaction of block and treatment within room and sex (animal term) and error. MF counts were natural log transformed prior to modeling, and back-transformed least squares means and corresponding confidence intervals were reported by treatment and time point.

For sarolaner and moxidectin, plasma concentration means and 90% confidence intervals were calculated for each treatment and time point using the statistical model described above. A natural log transformation was applied to plasma concentration data prior to modeling, and back-transformed least squares means and corresponding confidence intervals were reported by treatment and time point. For sarolaner and moxidectin estimates of the pharmacokinetic (PK) parameters C_max_, t_max_, AUC_0-672 h_ and t_1/2_ were made using non-compartmental methodology. For pyrantel C_max_, t_max_ and AUC_0-24 h_ were estimated. The means and confidence intervals for the PK parameters were calculated using a similar general linear mixed model for repeated measures as described above with dose period replacing time in the statistical model. AUC and C_max_ were natural log transformed prior to modeling, and back-transformed least squares means and corresponding confidence intervals were reported by treatment.

For the repeated measures models described above, if the treatment by sex by time interaction was significant at the 5% level of significance, then no further testing was done. If the sex by treatment by time interaction was not significant and the treatment by sex or time interaction was significant at the 10% level of significance, then treatment LS means were calculated, and pairwise comparisons between control and treatments were performed at the unadjusted 10% level of significance, within each sex if the treatment by sex interactions was significant (*P* ≤ 0.10) or within time point if the treatment by time interaction was significant (*P* ≤ 0.10) or time point if the treatment by time interaction was significant (*P* ≤ 0.10). If none of the interactions involving treatment were significant and the treatment main effect was significant at the 10% level of significance, then the treatment LS means were calculated, and pairwise comparisons between control and treatments were performed at the unadjusted 10% level of significance. If no treatment related effects were significant, no treatment comparisons were performed.

## Results

### Clinical signs

Clinical signs observed are summarized by treatment group in Table 2. Fever occurred in two treated animals (one dog in the 1× group, one dog in the 3× group) on Day 1 of the study. Both observations of fever resolved by Day 2 without medical intervention. Emesis was observed at low frequency in all treatment groups, including dogs in the control group, throughout the study. Minor sporadic decreases in weekly food consumption were observed in dogs in the 1× and 3× treatment groups, but there were no correlating changes in body weight. No hypersensitivity reactions (defined as anaphylaxis, shock, collapse, respiratory distress, depression or fever) were identified in any dog at any time point. Abnormal stool (soft stool or mucoid diarrhea or hemorrhagic colitis) was observed in two dogs in control group, four dogs in 1× group and seven in 3× group. This observation appeared to be treatment related. In all cases the effects observed were transient and the condition resolved without treatment.

**Table 2:** Clinical signs by treatment group (control group, 1× treatment group; 3× treatment group): number of animals affected and total number of observations Days 0–84

Clinical sign	Control		1×		3×	
	# Dogs	# Obs	# Dogs	# Obs	# Dogs	# Obs
Abnormal stools	2	3	4	23	7	23
Decreased appetite	0	0	4	5	5	6
Fever (> 104 °F/40 °C)	0	0	1	1	1	1
Emesis	3	3	2	3	4	7

### Microfilarial counts

**Fig. 1:**
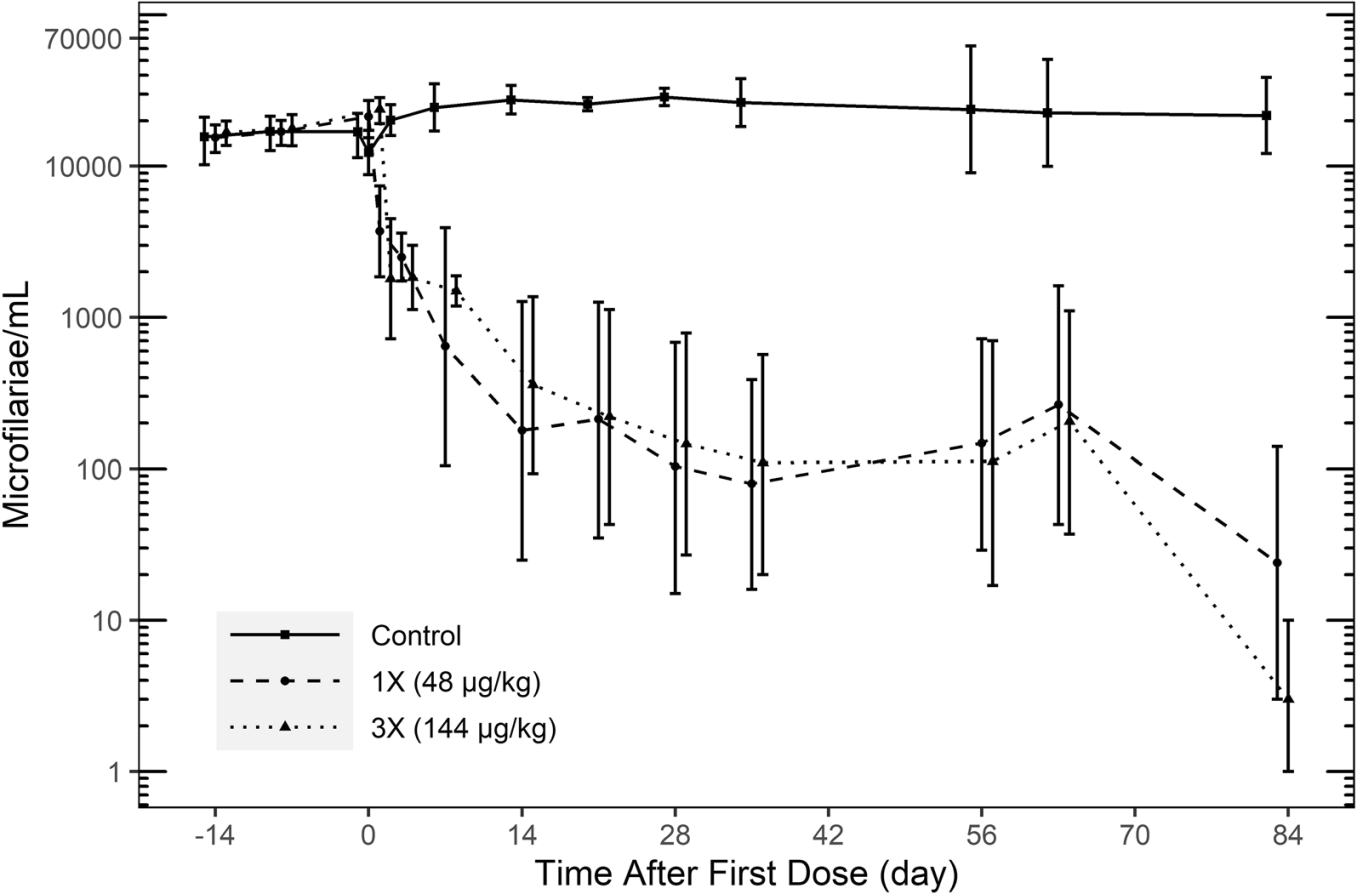
Mean microfilariae (MF) count/ml for each treatment by modified Knott’s method. All study dogs were evaluated for MF on Days -14, -8, 0 (pre-dosing), 1, 3, 7, 14, 21, 28 (pre-dosing), 35, 56 (pre-dosing), 63 and 83. A significant reduction (*P* < 0.10) in MF was observed in both 1× and 3× treatment groups at all time points following dosing

MF counts were performed throughout the study (Table 3). Mean MF counts (least square means) were significantly lower in both treated groups compared with the control group at every time point after Day 0 (*P* ≤ 0.016). The mean number of circulating microfilariae (MF) counts in the control group increased from 12,000/ml at study start (Day 0) through Day 28 to > 20,000/ml and remained above 20,000/ml for the remaining duration of the study. The least squares means of circulating MF were reduced by 69.8% on Day 1 and 97.4% on Day 7 for the 1× group and continued to remain > 99% lower relative to the control group for the remainder of the study. Similarly, least squares mean of circulating MF were reduced by 85.3% on Day 1 and 93.9% on Day 7 for the 3× group and continued to remain > 98% lower relative to the control group for the remainder of the study (Fig. 1).

**Table 3:** Least square means microfilariae count (counts/ml) for post-treatment time points

Study day	1	3	7	14	21	28	35	56	63	83
Control	12,289	20,149	24,440	27,462	25,695	28,659	26,342	23,717	22,519	21,622
1×	3711*	2503*	644*	179*	212*	104*	80*	147*	265*	24*
% Reduction	69.8	87.6	97.4	99.3	99.2	99.6	99.7	99.4	98.8	99.9
3×	1803*	1833*	1494*	359*	221*	146*	110*	112*	205*	3*
% Reduction	85.3	90.9	93.9	98.7	99.1	99.5	99.6	99.5	99.1	> 99.9

^*^Significant at two-sided alpha = 0.10 (*P* ≤ 0.10)

### Adult worm counts

At the end of the study, the mean number of implanted adult worms recovered was < 10 per sex in all groups with 90%, 85% and 75% of live adult heartworms recovered in control, 1× and 3× treatment groups, respectively (Table 4). No dead adult worms were found in the control group. Four (3 female and 1 male) dead adult worms were found in two dogs in the 1× group. Three (1 female and 2 male) dead adult worms were found in three dogs in the 3× group. All dogs in all treatment groups remained positive for heartworm antigen throughout the study.

**Table 4:** Recovery of total, live and dead heartworm counts on Day 84

Animal	Treatment	Total number of worms recovered	Number of adult males		Number of adult females	
			Live	Dead	Live	Dead
3,128,025 (M)	Control	13	4	0	9	0
3,128,972 (M)	Control	19	10	0	9	0
6,533,043 (M)	Control	19	10	0	9	0
6,533,116 (M)	Control	18	8	0	10	0
6,532,535 (F)	Control	19	9	0	10	0
6,532,578 (F)	Control	16	9	0	7	0
6,532,675 (F)	Control	18	8	0	10	0
6,532,918 (F)	Control	20	10	0	10	0
6,532,152 (M)	1X	15	8	0	7	0
6,532,187 (M)	1X	17	6	0	11	0
6,532,217 (M)	1X	18	11	0	7	0
6,532,241 (M)	1X	17	8	0	9	0
3,124,127 (F)	1X	19	9	0	10	0
3,124,763 (F)	1X	18	9	0	9	0
6,532,501 (F)	1X	15	7	1	5	2
6,532,519 (F)	1X	18	9	0	8	1
3,127,380 (M)	3X	9	1	0	8	0
6,532,560 (M)	3X	15	9	0	5	1
6,532,641 (M)	3X	10	4	0	6	0
6,533,027 (M)	3X	17	8	0	9	0
6,532,250 (F)	3X	19	10	0	9	0
6,532,764 (F)	3X	17	8	1	8	0
6,533,159 (F)	3X	18	8	0	10	0
6,533,167 (F)	3X	17	8	1	8	0

### Pharmacokinetic data

**Fig. 2:**
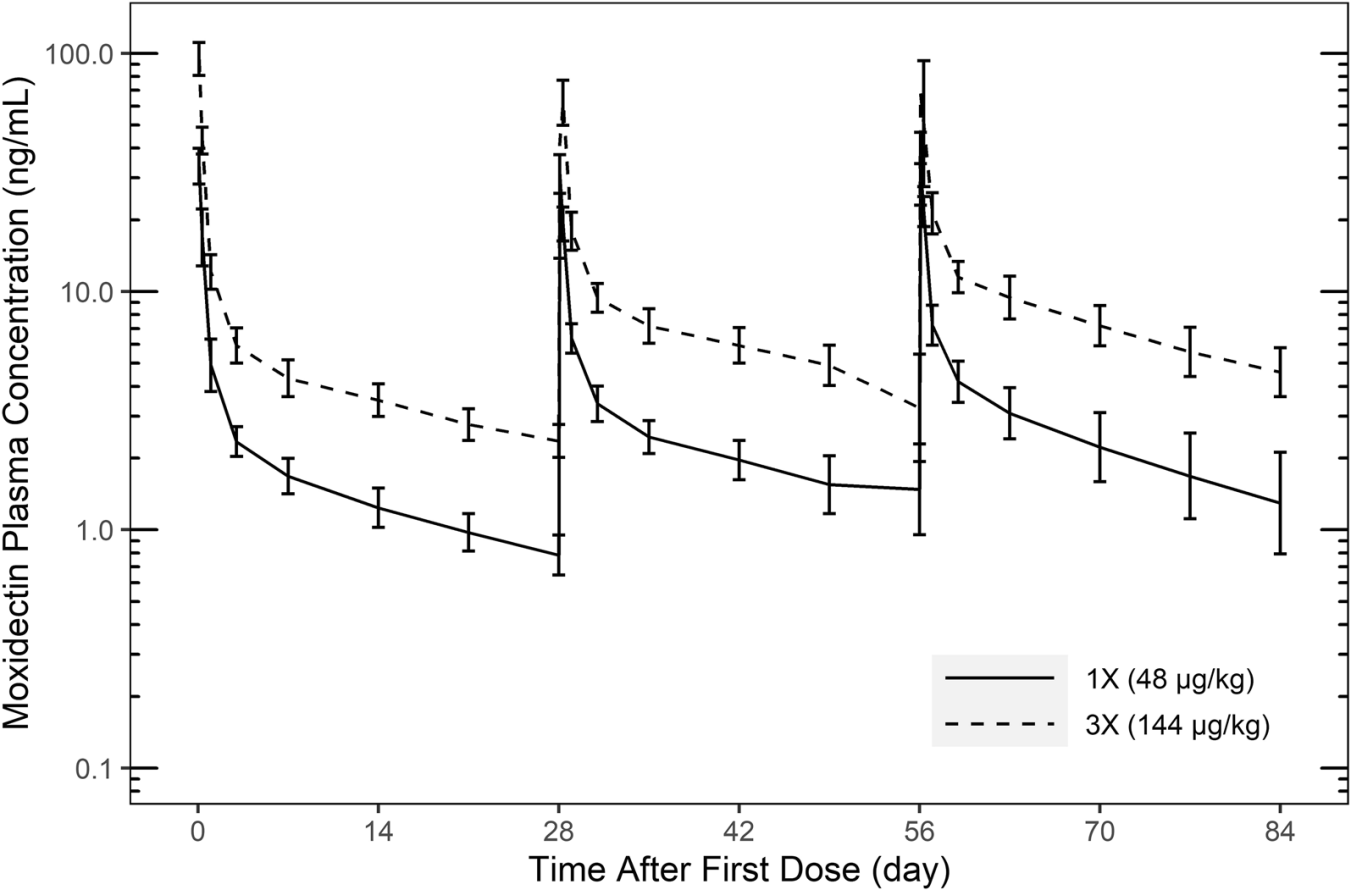
LS mean (± 90% CI) moxidectin plasma concentration following each of 3 monthly (28-day interval) doses of Simparica Trio

As expected, the pharmacokinetic analysis demonstrated the LS mean plasma concentration in the 3× group was approximately three times higher than in the 1× group for both moxidectin (Fig. 2) and sarolaner. Pyrantel had very low bioavailability and very high variability, but plasma concentrations did show an increased level with dose. Following each dose, the moxidectin and sarolaner AUC and C_max_ had expected dose-related increases. Although not formally tested, the increases appear to be close to dose proportional. Moxidectin and sarolaner show accumulation in dose-normalized AUC_0-672 h_. The accumulation ratios based on AUC_0-672 h_ from dose 1 to dose 3 for moxidectin and sarolaner were approximately 1.7.

## Discussion

In this study, the safety of a novel combination of sarolaner, pyrantel and moxidectin (Simparica Trio, Zoetis) in dogs experimentally infected with adult *D. immitis* and with circulating microfilariae was evaluated. When used per label, a single dose of Simparica Trio in the commercially available formulation delivers a dose of moxidectin between 24–48 µg/kg. Treatment of heartworm-positive dogs with 1× and 3× the maximum label dose of Simparica Trio was well tolerated with treatment-related observations limited to occasional abnormal stools (e.g. soft stool, diarrhea), decreased appetite and, rarely, fever.

### Microfilaricidal activity

Moxidectin administration resulted in significant reduction of MF counts at both doses of 48 µg/kg (1×) and 144 µg/kg (3×) in dogs infected with adult *D. immitis* (Fig. 1). The mean MF count in the control group increased from 12,000/ml at study start (Day 0) to > 20,000/ml at Day 28 and remained > 20,000/ml for the duration of the study. The mean number of circulating MFs were reduced by > 90% on Day 3 for the 144 µg/kg (3×) group and by Day 7 for the 48 µg/kg (1×) group compared with controls and remained > 98% compared to the control group for the remainder of the study.

The only relevant clinical sign observed was fever in two dogs approximately 24 h after dosing with Simparica Trio. A single 1× dog had a body temperature of 104.1 °F (40.0 °C) on the day after treatment. This dog’s MF count was 22,000/ml just prior to treatment with Simparica Trio, falling to 3900/ml the following day which corresponded to a > 80% reduction. A second dog, in the 3× group, was described as “subdued” and had a body temperature of 105.4 °F (40.7 °C) on the day after treatment. This dog had a pre-treatment body temperature of 104.5 °F (40.3 °C) possibly indicating that the dog was reacting to its high microfilaremia prior to dosing with Simparica Trio. This dog’s MF count was 27,000/ml just prior to treatment and fell to 1500/ml the following day, a 93% reduction. Both dogs cleared all MF by 1 (1×) and 3 (3×) weeks after the first treatment. The fever exhibited by these dogs could be attributed to the immune response to the dying microfilariae after they had been exposed to moxidectin. It is hypothesized that the ML activity on glutamate gated chloride ion channels present in the excretory/secretory pore of microfilariae inhibits the secretion of immunomodulatory molecules, thereby allowing the host immune response to clear microfilariae [12].

By the end of the study after three treatments with Simparica Trio, three dogs in 1× treatment group and two dogs in 3× treatment group had dropped to zero MF count. The two dogs discussed above exhibiting fever were the only two dogs with complete clearance of MF prior to the last data collection point. Although other dogs had higher MF counts pre-dose, they did not exhibit fever after dosing with Simparica Trio. This may be due to individual variability in immune response or because the mild fever was of such a short duration that it was not present at the times of recording.

Overall, despite a rapid decline in MF counts in both treatment groups, which has also been observed with other ML-containing heartworm disease preventives, no severe or anaphylactic-like adverse reactions akin to those observed with the previously used daily heartworm preventive drug, diethylcarbamazine, occurred [6].

### Adulticidal activity

The rapid killing of adult heartworms in dogs could result in severe pulmonary thromboembolism or death [13]. There were no clinical observations attributable to rapid adulticidal activity in this study. Occasionally, as a result of surgical transplant procedure, worms may be injured and subsequently die and eventually be absorbed by the body, and this was indicated by recovering < 20 worms (live or dead) from some animals in the control group. The recovery of the majority of adult male and female live worms in both 1× (48 µg/kg) and 3× (144 µg/kg) groups, however, indicates there was no rapid kill of worms and there were no clinical signs consistent with severe pulmonary thromboembolism: cough, dyspnea, exercise intolerance, collapse, hemoptysis and death. There were more dead worms in the Simparica Trio-treated groups than in the control group, indicating that moxidectin may have had some minor adulticidal activity, but with no clinical sequelae.

## Conclusions

This study demonstrated that dogs that had been previously infected with ML-susceptible adult *D. immitis* (ZoeKY isolate) and subsequently treated with Simparica Trio at 1× and 3× the maximum recommended label dose, three times at 28-day intervals, was well tolerated. Simparica Trio caused a rapid but clinically benign reduction of microfilariae counts in both treatment groups with minimal effects on adult worms.

## Data Availability

Not applicable.
